# MOOC construction for life education in Chinese universities: an analytical study

**DOI:** 10.3389/fpubh.2025.1569881

**Published:** 2025-07-02

**Authors:** Fengling Xu, Junqing Zhang, Qiqi Zhou, Shaoyu Mou

**Affiliations:** School of Nursing, Chongqing Medical University, Chongqing, China

**Keywords:** life education, MOOC, online courses, curriculum development, quality assessment

## Abstract

**Background:**

With the advancement of the Chinese “Internet Plus” paradigm, life education has expended through the implementation of Massive open online courses (MOOCs). However, limited research has focused on the present condition and attributes of the building of Chinese life education MOOCs.

**Objectives:**

To systematically analyze the current status and attributes of life education MOOCs in China, assess the quality of the courses, and give a reference for the development of life education MOOCs in the country.

**Methods:**

We select China’s online education platform and utilize the search terms “Health OR Life OR Death OR Psychology OR Nutrition OR Exercise OR Well-being OR Love OR Ethics” to identify MOOCs pertinent to life education. Analyze and summarize the courses about platforms, schools, regions, objectives, contents, assessments, credit hours, number of sessions and number of learners. The quality of the course was assessed utilizing Quality Matters (QM).

**Results:**

In China, 129 life education MOOCs were provided in six online education platforms. The quantity of courses originating from “double first-class” universities, general universities and higher vocational universities was 44, 40, and 45, respectively. The quantity of courses from eastern regions was 106, while from western regions it was 23. The course contents covered three domains: life education, death education, and life value education. Credit hours, course sessions and the number of course learners exhibit statistically significant differences among regions and school types (*p* < 0.05). 47.3% of the QM scores were 60–84%, indicating subpar overall course quality. 19 life education MOOCs were provided in foreign platforms Coursera and edX, with 52.6% having course duration of less than 16 h and 47.4% catering to over 100,000 participants.

**Conclusion:**

Chinese life education MOOC is resource-rich and has a substantial learner base. However, issues persist, including inconsistent course quality, limited personalization of course content, and significant disparities between eastern and western regions. In the future, Chinese life education MOOC should align with the development in Artificial Intelligence (AI) technology, address the disparities between Eastern and Western areas, and integrate with worldwide platforms.

## 1 Introduction

With the emergence of the “Internet Plus” model and the extensive utilization of contemporary information technology in China, medical online education has developed rapidly, resulting in the birth of many online educational practice modalities (1). In 2008, Dave Cormier initially introduced the notion of Massive Open Online Courses (MOOCs) (2). Wikipedia defines MOOC as “a type of course delivered online, open to an unlimited number of participants, allowing learners to access free courses and obtain corresponding certificates of completion (3).” MOOC consists of four fundamental components: (1) Massive: MOOC encompasses a substantial quantity of learners and educators, including all societal sectors with a desire for knowledge; (2) Open: MOOC education is characterized by its accessibility, allowing users to learn at any time and location, with courses offered at no cost; (3) Online: MOOC courses are delivered via the Internet to offer online resources; (4) Course: MOOC provides a comprehensive course framework, encompassing learning objectives, content, assessment, and certification as a foundation for course engagement (2).

Massive open online courses rely on the Internet online platforms to provide open and varied courses for learners globally (2). The advent of MOOCs has created a novel possibility for the advancement of medical education, transcending temporal and spatial constraints, and providing students with a plethora of systematic and professional materials to select from (4). Against the background of rapid economic and social development, constant changes in the natural environment, and intensifying social competitiveness, public health awareness is gradually increasing, prompting academics to focus increasingly on life education (5).

In the 1960s, in response to adolescents’ suicidal, self-injurious and other maladaptive behaviors, American researcher James Donald Walters pioneered the concept of life education and developed a corresponding school life education curriculum system (6). Since then, life education has progressively expanded worldwide. In recent years, college students have had considerable mental health issues, and they possess an ambiguous comprehension of their perspectives on life and death (7). Currently, life education in Chinese colleges and universities is inadequate, and the demand for life and death education among college students, particularly those in medical fields, is rising, necessitating an urgent enhancement of life education for these students (8). Consequently, various types of college life education have arisen as MOOCs, offering students an expanded array of learning opportunities. The development of life education programs in medical institutions has positively influenced medical students’ perspectives on life and death, fostering their comprehensive growth (8). At present, the majority of research on life education, both in China and internationally, consists of theoretical analyses and value reflections (9), with limited reports addressing the current state and characteristics of life education MOOC development. This study investigated the current status of life education MOOCs in universities to provide reference for enhancing the development of online life education courses and facilitating the dissemination of high-quality teaching resources.

## 2 Methods

### 2.1 Study design

This study gathers data from major online course platforms in China and internationally to compare and analyze the current status of life education MOOC development in China. The studies were approved by Ethics Committee of the Chongqing Medical University (2023070), on 3 September 2023. The studies were conducted in accordance with the local legislation and institutional requirements.

### 2.2 Participants

**Figure 1:**
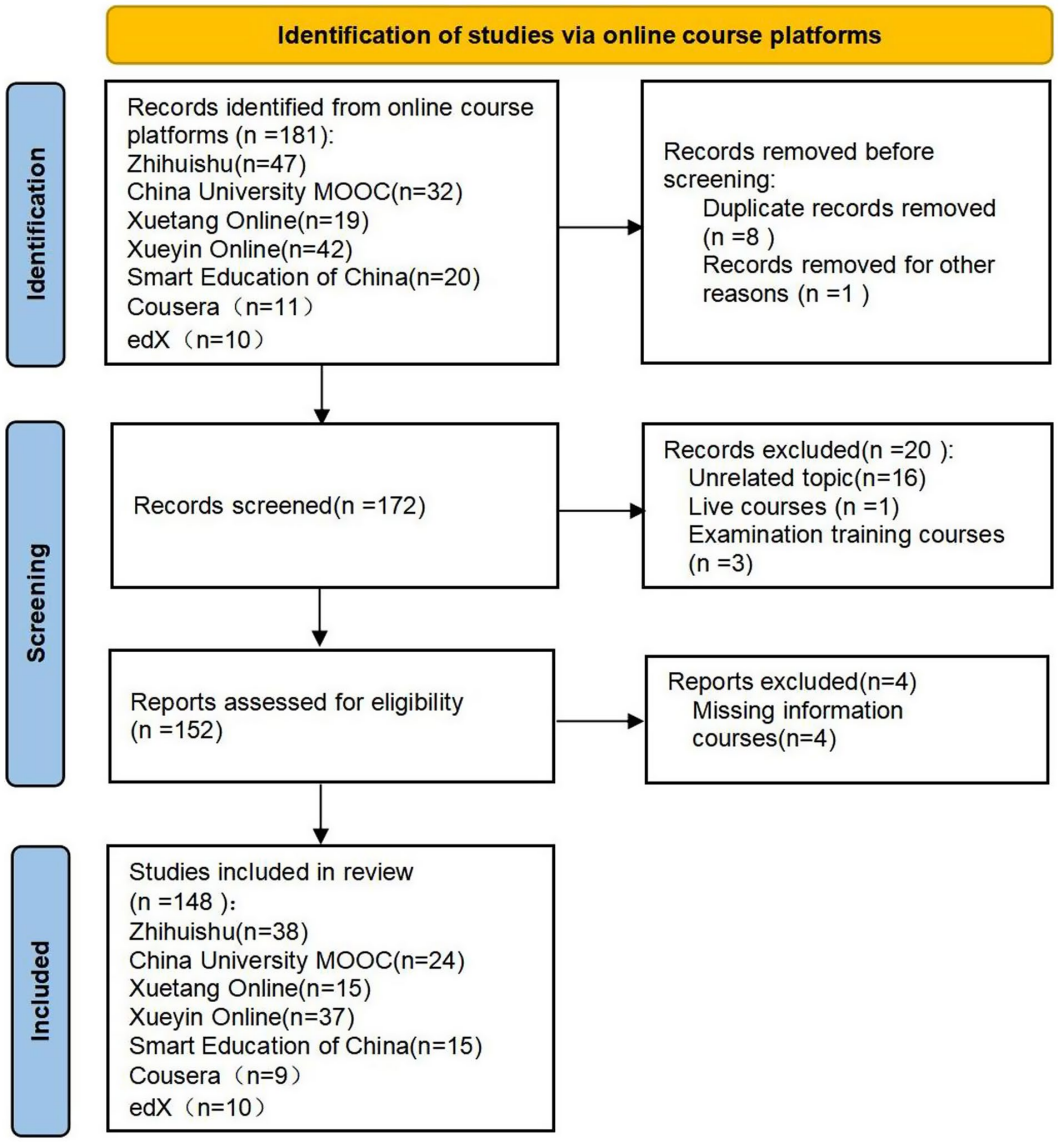
The course screening flowchart.

Based on the “China Digital Education Top 100 List in 2023”^1^ and the historical development of online education platforms in Chinese higher education (10), the major Chinese and international higher online education platforms were identified by evaluating platform registrations, subject categories, course offerings, and the representation of colleges and universities on these platforms. Five Chinese online education platforms, namely Zhihuishu^2^, Xueyin Online^3^, China University MOOC^4^, Smart Education of China^5^, and Xuetang Online^6^, were chosen, along with the international online education platforms Coursera^7^ and deX^8^ for course exploration. Inclusion criteria for online courses: (1) MOOC courses; (2) developed by higher education institutions; (3) freely accessible and open courses; (4) comprehensive course design. Exclusion criteria: (1) live courses; (2) examination training courses; (3) duplicate courses. The course screening flowchart is shown in Figure 1.

### 2.3 Methods

In contemporary society, life education for college students has emerged as a prominent and significant topic, encompassing a diverse array of educational content, including life awareness education, death education, and survival skills education (11). Therefore, we explored life education courses using the online education platform, employing the search terms “Health OR Life OR Death OR Psychology OR Nutrition OR Exercise OR Well-being OR Love OR Ethics” to identify relevant courses. The search deadline was April 5, 2024. To ensure the objectivity and accuracy of the data, one expert with a decade of higher education teaching experience, who has contributed to the development of online education, and two master’s degree students in the relevant field were engaged in data collection and evaluation.

### 2.4 Outcome indicators

Compilation of course details: Data on course platforms, course schools, course regions, course contents, course evaluations, course credit hours, number of course sessions and number of learners was collected through the online education platform system for generalization and analysis.

Criteria for Evaluating Course Quality: Quality Matters (QM)^9^ is one of the most popular and widely used curriculum quality assurance frameworks in the United States (12). It focuses on improving the quality of online courses at the K-12, higher education, and professional education sectors, comprising eight peer-reviewed criteria: (1) Course overview and introduction; (2) Learning objectives; (3) Assessment and measurement; (4) Instructional materials; (5) Learning activities and learner interaction; (6) Course technology; (7) Learner support; (8) Accessibility and usability. Each criterion encompasses more precise sub-criteria. QM scores range from 0 to 101, with each entry assigned a variable score of 1 to 3 points, awarded if the criterion is fulfilled and 0 if not. A total score of at least 85 is required to pass the review of a course.

### 2.5 Statistics

Statistical analyses were conducted with SPSS 26.0 software. Categorical variables were represented as frequencies and percentages. Comparisons of differences between the two cohorts were conducted using chi-squared tests, with *P*<0.05 as statistically significant.

## 3 Results

### 3.1 Origin and distribution of courses

In China, 129 life education MOOCs were available across five online education platforms, comprising 44 (34.1%) from “double first-class” universities, 40 (31.0%) from general universities, and 45 (34.9%) from higher vocational universities. The “Heihe-Tengchong line” divides China into eastern and western regions, with 106 courses (82.6%) were developed by universities in the eastern region, and 23 courses (17.8%) were developed by universities in the western region. The distribution of life education MOOCs is shown in Table 1.

**Table 1:** Distribution of life education MOOCs [*N* = 129, courses (%)].

Terms	Measures	Regional distribution		Type of university			Total
		Eastern	Western	“Double first-class”	General	Higher vocational	
Total		106 (82.2)	23 (17.8)	44 (34.1)	40 (31.0)	45 (34.9)	129
Credit hours	<16	18 (17.0)	5 (21.7)	13 (29.5)^ab^	6 (15.0)	4 (8.9)	23 (13.2)
	16–32	55 (51.9)	11 (47.8)	15 (34.1)	26 (65.0)	25 (55.6)	66 (55.0)
	>32	33 (31.1)	7 (30.4)	16 (36.4)	8 (20.0)	16 (35.6)	40 (31.8)
*X^2^*		0.293		12.028			
*P*		0.864		0.017			
Course sessions	1–4	22 (20.8)	2 (8.7)	3 (6.8)^cd^	6 (15.0)	15 (33.3)	24 (18.6)
	5–10	57 (53.8)	15 (65.2)	22 (50.0)	27 (67.5)	23 (51.1)	72 (55.8)
	11–21	27 (25.5)	6 (26.1)	19 (43.2)	7 (17.5)	7 (15.6)	33 (25.6)
*X^2^*		2.198		18.353			
*P*		0.333		0.001			
Number of course learners (10,000)	<1	35 (33.0)	6 (26.1)	18 (40.9)	11 (27.5)	12 (26.7)	41 (31.8)
	1–10	56 (52.8)	15 (65.2)	18 (40.9)	25 (62.5)	28 (63.6)	71 (55.0)
	>10	15 (14.2)	2 (8.7)	8 (18.2)	4 (10.0)	5 (11.4)	17 (13.3)
*X^2^*		1.278		5.456			
*P*		0.528		0.243			

a: Compared with general universities *X*^2^ = 8.025, *p* = 0.018. b: compared with higher vocational universities *X*^2^ = 7.094, *p* = 0.029; c: compared with general universities *X*^2^ = 7.094, *p* = 0.029; d: compared with higher vocational universities *X*^2^ = 13.551, *p* = 0.001.

The international platforms include Coursera and edX, with the 19 courses originating from prestigious institutions such as Yale University, Harvard University and Peking University, as shown in Table 2.

**Table 2:** Life MOOC offerings on Coursera and edX (N = 19).

Course name	Name of the school/company	Course content	Course platforms	Course credit hours	Course learners (10,000)	Course level	Course rating(Total score = 5)
1. Life 101: Physical and Mental Self-Care	University of California, Irvine	Self-care; Emotions and stress; Nutrition and health; Positive thinking and emotional intelligence; Exercise and sleep;	Coursera	15	1.6	Elementary	4.8
2. Finding Meaning and Purpose in Life: Living for What Matters Most	University of Michigan	Life goals and growth	Coursera	11	25.5	Elementary	4.8
3. Life Safety and Rescue	Shanghai Jiao Tong University	Health assessment; Sports protection; Wilderness survival; Accident management	Coursera	22	0.3	/	4.4
4. Healthy Practices: Nutrition, Physical Activity, and Community and Family Engagement	University of Colorado System	Nutrition and Health; Exercise and Health; Community, Family, and School Health Sessions	Coursera	9	2.6	Elementary	4.7
5. Positive Psychiatry and Mental Health	The University of Sydney	Positive mental health; Body and mind; Love and work; Mental illnesses	Coursera	19	30.6	/	4.8
6. The Science of Happiness	Yale University	Recognizing Happiness; Meeting the Challenge	Coursera	19	482.0	/	4.9
7. Mindfulness and Well-being Specialization	Rice University	Mindfulness and well-being	Coursera	60	1.2	Elementary	4.9
8. The Science of Adolescent Well-Being	Yale University	Perceptions of happiness; Behaviors of happiness; Pursuit of happiness	Coursera	12	16.5	Elementary	4.9
9. A Happy and Fulfilling Life	Indian School of Business	Misconceptions of happiness; Exercises for happiness; Love and devotion; Positive thinking and happiness	Coursera	27	48.7	/	4.8
10. The Path to Happiness: What Chinese Philosophy Teaches us about the Good Life	Harvard University	Ancient Chinese philosophy and self-awareness	deX	20	13.8	/	4.7
11. Nutrition and Health: Macro-nutrients and Over-nutrition	Wageningen University	Nutrient composition; Nutrition and health relationships	deX	63	23.7	/	4.5
12. Nutrition and Healthy Living	University of California, Riverside	Nutrient composition; Nutrition and health relationships	deX	9	<1.0	/	4.5
13. Talk to me’: Improving mental health and suicide prevention in young adults	Curtin University	Factors in poor mental health; Strategies to improve mental status	deX	15	2.3	/	4.6
14. Mental Health, Mindfulness, and Self-Care	University of Cape Town	Mental problems; Mental enhancement exercises	deX	12	<1.0	/	4.7
15. The Science of Happiness	University of California, Berkeley	The Meaning of Happiness; Ways to Enhance Happiness; Enhancing Happiness with Positive Thoughts	deX	50	59.7	/	4.7
16. Finding Purpose Across Your Life	University of California, Berkeley	The meaning of purpose in life; Ways to achieve purpose in life	deX	9	<1.0	/	4.8
17. Building Personal Resilience: Managing Anxiety and Mental Health	Harvard University	Mentall adaptation and mental health	deX	9	12.7	/	4.6
18. Mental Health and Nutrition	University of Canterbury	Diet, nutrition and mental health	deX	32	8.6	/	4.7
19. Spiritual Competency Training in Mental Health	University System of Maryland	Clinical Practice; Mental Health; Spirituality	deX	13	<1.0	/	4.3

### 3.2 Course operation

The course operation contains three components: course credit hours, number of course sessions and number of course learners. In China, among the 129 life education MOOCs, course credit hours varied from 5–80 h, with 66 (55.0%) courses offering 16–32 h, the school type significantly influenced course (*p* < 0.05). Of the 129 life education MOOCs, 1–21 course sessions were offered, with 72 courses (55.8%) featuring a focus of 5–10 course sessions, equating to 2 periods every year, the school type significantly influenced course sessions(*p* < 0.05). Regarding the number of course learners, 71 courses (55.0%) had between 10,000 and 100,000 learners, while 17 courses (13.3%) exceeded 100,000 learners, as illustrated in Table 1.

Among the 19 life education MOOCs available on Coursera and edX, 10 courses (52.6%) offer fewer than 16 credit hours, 6 courses (31.6%) provide 16–32 credit hours, and 3 courses (15.8%) exceed 32 credit hours. Regarding course participants, there are 5 courses (26.3%) with fewer than 10,000 participants, 5 courses (26.3%) with 10,000 to 100,000 participants, and 9 courses (47.4%) with more than 100,000 participants, as shown in Table 2.

### 3.3 Assessment of fundamental components of courses

According to pedagogical theories, the fundamental components of the course encompass four aspects: objectives, contents, process, and evaluation (13). This research studies three areas of life education MOOC data in China and internationally: objectives, content, and assessment.

#### 3.3.1 Objectives and contents of the course

**Figure 2:**
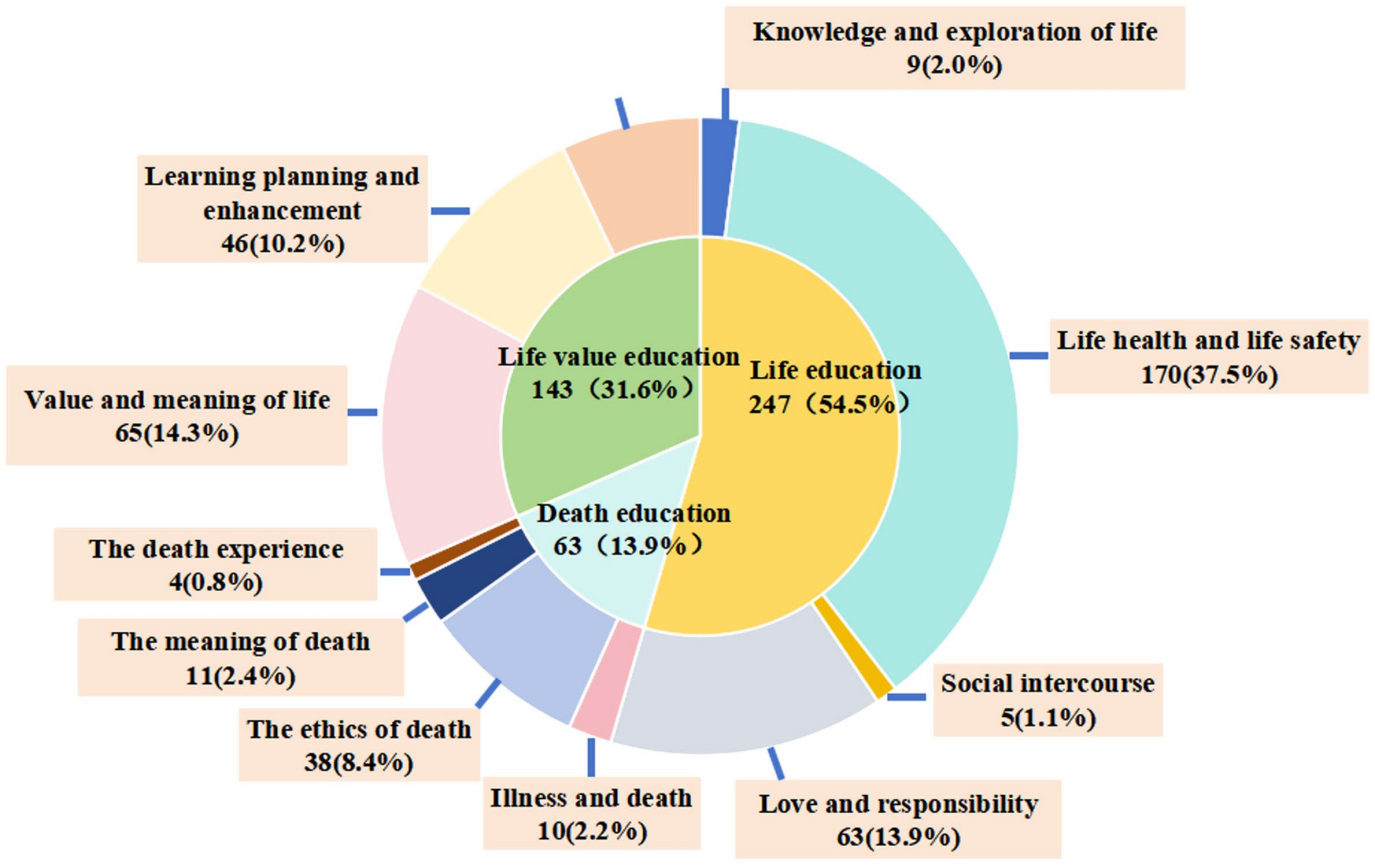
Pie chart of content distribution and frequency of themes in Chinese life education MOOCs [*N* = 453, times (%)].

Chinese life education MOOCs are structured on the central topic of “respecting and cherishing life” (14), encompassing 3 primary themes, 11 secondary themes and 453 keywords in this study. Each secondary theme encompasses various keywords, indicating that each course addresses an extensive array of content and subjects, as shown in Table 3. Of the primary themes, life education at 54.5%, life value education at 31.6%, and death education at 13.9%, as shown in Figure 2, with secondary themes exhibiting varying frequency distributions.

**Table 3:** Chinese life education MOOC topic content for different levels.

Primary themes	Secondary themes	Keywords
1. Life education	1. Knowledge and exploration of life	Understanding life; Natural symbiosis; Origin and evolution of life
	2. Life health and life safety	Mental health; Disease prevention; Physical activity; Nutritional management; Emergency evacuation; First Aid knowledge
	3. Social intercourse	Humanistic care; Interpersonal communication; Wisdom in the world
	4. Love and responsibility	Marriage; Parents; Brothers; Children; Friends; Love; Career; Social responsibility
2. Death education	5. Illness and death	Sickness and aging; Dying and death
	6. The ethics of death	Hospice care; Fertility management; Organ transplants; Human experimentation; Euthanasia; Sexually transmitted diseases
	7. The meaning of death	Recognizing death; Living toward death; Transcending death
	8. The death experience	Near-Death experiences; Funeral culture; Bucket lists
3. Life values education	9. Value and meaning of life	Respect life; Cherish life; Love life
	10. Learning planning and enhancement	Adaptability; Planning; Advancement; Learning
	11. Personality development and advancement	Optimize personality; Transcend ego; Realize life

The 19 courses offered by the Coursera and edX platforms, the topics are similar to those in China, with primary themes including “the meaning of life, life safety, physical and mental health, nutrition and health, and happy life.” However, the material covered in each course is very specialized, and numerous comprehensive and advanced courses have been developed, as shown in Table 2.

#### 3.3.2 Course assessment

**Figure 3:**
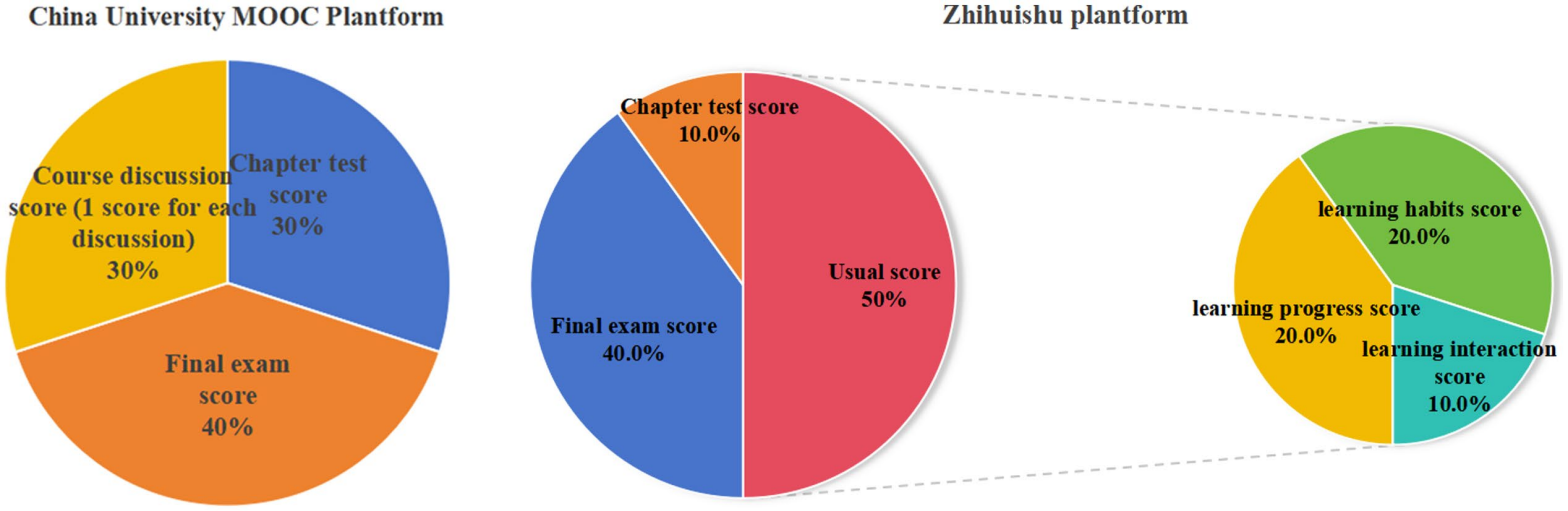
Course assessment standard for Zhihuishu and China University MOOC platforms.

The Life education MOOC course is an online course, with assessment standard predominantly established by the platform. This includes the usual grades, chapter test scores, final examination results. Figure 3 illustrates the calculation formula of the Zhihuishu and China University MOOC platform, the percentage of grades of each platform is slightly different. Upon fulfilling the course requirements and assessment criteria, students may submit for certification, which will automatically produce the electronic form of the certificate.

### 3.4 Quality matters results

All 129 life education MOOCs in China received QM scores below 85, hence failing to satisfy the QM quality review passing standard. 61 courses (47.3%) received ratings ranging from 60 to 84, while the remainder scored below 60, as seen in Table 4. Regarding platform distribution, 29 courses (22.5%) on Zhihuishu and 17 courses (13.2%) on the China University MOOC platforms achieved scores between 60 and 84 points. Meanwhile, 15 courses (11.6%) on the Smart Education of China platform scored below 60 points, with 9 courses scoring below 40 points. Additionally, this platform was deficient in essential QM scoring components, including course introduction and course materials. Despite 27 courses in Xueyin Online receiving lower grades, the portal provides comprehensive course descriptions, objectives, and target audience criteria for each course.

**Table 4:** Results of quality matter scoring of life education MOOC in China [*N* = 129, times (%)].

Course platforms	QM score (%)			
	≤39%	40–59%	60–84%	≥85%
Zhihuishu	0 (0.0)	9 (7.0)	29 (22.5)	0 (0.0)
China University MOOC	0 (0.0)	7 (5.4)	17 (13.2)	0 (0.0)
Xuetang Online	0 (0.0)	10 (7.8)	5 (3.9)	0 (0.0)
Xueyin Online	0 (0.0)	27 (20.9)	10 (7.8)	0 (0.0)
Smart Education of China	9 (7.0)	6 (4.7)	0 (0.0)	0 (0.0)
Total	9 (7.0)	59 (45.7)	61 (47.3)	0 (0.0)

The top five courses in China’s 129 life education MOOC rankings, according to QM rating, predominantly come from the Zhihuishu and China University MOOC platforms, primarily located in the eastern coastline region, with course levels mainly at the national and provincial tiers. The bottom 5 ranked courses mainly originated from higher vocational colleges and universities, and were mainly school-level courses, as shown in Table 5. The analysis of QM scores indicates that the top five courses fulfill the majority of the criteria, with the exception of “Dimension 7: Learner support,” “Dimension 1: Course overview and introduction” and “Dimension 2: Learning objectives.” Furthermore, the bottom 5 courses failed to enhance “Dimension 3: Assessment and measurement” and “Dimension 6: Course technology,” as shown in Table 6.

**Table 5:** Information on the top and bottom 5 life education courses among the 129 MOOCs in China.

Top 5 QM ratings				Bottom 5 QM ratings			
Course name	Course platform	Course source	Course level	Course name	Course platform	Course source	Course level
1. Mental Health Education for College Students	Zhihuishu	Hainan College of Economics and Trade; Hainan	National Level	1. Senior Sports and Health	Smart Education of China	Tangshan Institute of Industrial Technology; Hebei	University Level
2. Sports Safety and Health	Zhihuishu	Zhejiang University; Jiangsu	National Level	2. Medical Ethics	Smart Education of China	Hubei Medical College; Hubei	University Level
3. Medical Ethics	Zhihuishu	Shandong Second Medical University; Shandong	Provincial Level	3. Health Education and Health Promotion	Smart Education of China	Henan Medical College; Henan	University Level
4. Life Education and Life Growth	China University MOOC	Luoyang Normal College; Henan	University Level	4. Nursing Ethics and Health Regulations	Smart Education of China	Xiangyang Institute of Vocational Technology; Hubei	University Level
5. Food Nutrition and Food Safety	China University MOOC	Central South University; Hunan	National Level	5. Mental Health Education	Smart Education of China	Xingtai Medical College; Hebei	University Level

**Table 6:** QM scores of the Top 5 and bottom 5 life education courses.

Course Ranking	Dim-1									Dim-2					Dim-3						Dim-4					Dim-5				Dim-6				Dim-7				Dim-8						
	1	2	3	4	5	6	7	8	9	1	2	3	4	5	1	2	3	4	5	6	1	2	3	4	5	1	2	3	4	1	2	3	4	1	2	3	4	1	2	3	4	5	6	7
1	3	3	2	–	–	1	–	1	–	3	–	3	3	3	3	3	3	2	2	–	3	3	–	2	2	3	3	3	2	3	2	1	1	–	–	–	–	3	3	–	–	2	2	–
2	3	3	2	–	–	1	–	1	–	3	–	3	3	–	–	3	3	2	2	–	3	3	–	2	2	3	3	3	2	3	2	1	1	–	–	3	–	3	3	3	–	2	2	–
3	3	3	2	–	–	1	–	1	–	–	–	3	3	–	3	3	3	2	2	–	3	3	–	2	2	3	3	3	2	3	2	1	1	–	–	–	–	3	3	3	2	2	2	–
4	3	3	2	–	–	1	–	–	–	3	–	3	3	3	3	3	3	2	2	–	3	3	2	2	2	3	3	–	2	–	–	–	1	–	–	3	–	3	3	3	2	2	–	–
5	–	3	2	–	–	1	–	–	–	3	–	3	3	3	3	3	3	2	2	–	3	3	2	2	2	3	3	3	2	–	–	–	1	–	–	3	–	3	3	3	2	2	–	–
125	3	3	–	–	–	1	–	–	–	–	–	3	–	–	–	–	–	–	2	–	3	–	–	–	–	3	–	–	2	–	–	–	1	–	–	–	–	3	3	3	2	2	–	–
126	–	–	–	–	–	–	–	–	–	–	–	–	–	–	3	3	3	2	2		3	–	–	–	–	3	–	–	2	–	–	–	1	–	–	–	–	3	3	3	2	2	–	–
127	–	–	–	–	–	–	–	–	–	–	–	3	–	–	–	–	–	2	2	–	3	3	2	2	2	–	3	–	–	–	–	–	1	–	–	–	–	3	3	–	–			–
128	–	–	–	–	–	–	–	–	–	–	–	–	–	–	–	–	–	–	2	–	3	–	2	–	2	3	–	–	–	–	–	–	1	–	–	–	–	3	3	3	2	2	–	–
129	–	–	–	–	–	–	–	–	–	–	–	–	–	–	–	–	–	–	2	–	3	–	–	2	–	3	3	–	–	–	–	–	1	–	–	–	–	3	3	3	–	2	–	–

Marked with “1 2 3”are satisfied entries and points earned, marked with “–” are unfulfilled entries with 0 points.

The QM compliance rate for 19 foreign courses is 80%. Most courses on the Coursera platform are categorized by learning levels, while the edX platform features participant ratings and evaluations, both of which adhere to QM scoring standards and offer guidance for learners in selecting the most appropriate courses.

## 4 Discussion

### 4.1 Life education MOOCs have achieved developments in China

In recent years, China attaches great importance to the development of the “Internet plus” model, with colleges and universities serving as the primary platforms for MOOC development, demonstrating greater efficacy in enhancing student learning compared to conventional teaching methods (15). This study shows the extensive availability of online course resources for life education in China, sourced from general colleges and universities and higher vocational colleges nationwide. Notably, platforms like Zhihuishu and China University MOOC aggregate resources from prestigious institutions, including Sichuan University and Shandong University, thereby offering high-quality educational materials for the advancement of life education in higher education institutions. The analysis found that the number of course learners surpassed 100,000, representing 13.3%, while the number of courses offered constituted 81.4% of the total, indicating that life education has become a significant component of China’s online education. This suggests that the MOOC model effectively addresses students’ learning requirements, and the platform’s infrastructure aligns with the technological and content needs of learners (16), consistent with China’s developmental trend to leverage the advantages of online education and enhance the accessibility of educational resources (17).

Nonetheless, disparities exist in the quantity of course sessions and the number of participants within the region housing 129 MOOC institutions, as well as in the type of institution (*p* < 0.05). The MOOC for life education in “double first-class” universities was established earlier, attracting a larger cohort of learners and achieving relatively high quality evaluation scores for QM courses. In contrast, higher vocational colleges and universities exhibit a deficiency in online course resources, with fewer courses, limited duration, and a smaller number of participants, indicating an imbalance in the distribution of MOOC resources for life education in China (18). This study reveals a significant disparity in the quality of resources and course utilization between eastern and western institutions, as well as between undergraduate and higher vocational institutions. This discrepancy hampers the effective utilization of MOOC resources in life education and impedes the widespread adoption of life education, reflecting the uneven distribution of health education resources across eastern and western China (19, 20). In the future, institutions in the East and West should adhere to the principle of “resource sharing and wisdom coexistence” to collaboratively develop standardized and exemplary online courses, thereby maximizing the dissemination and application of high-quality life education resources and ensuring equitable access to life education for all individuals.

### 4.2 Chinese life education MOOCs still need to learn from international platforms

The Coursera platform, established by Stanford University, was officially launched in 2012 to offer free online courses, followed by the edX platform, co-founded by Massachusetts Institute of Technology (MIT) and Harvard University in 2013 (21). The life education MOOCs in both platforms cover English and Chinese learning resources, and developed by globally renowned universities. The courses are exceptionally appealing and widely employed, drawing involvement from numerous learners and educational institutions. All life education MOOCs on Coursera have open student rating and commenting modules, which offer precise learning recommendations and a transparent evaluation method, hence facilitating effective learning and ongoing course enhancement for learners.

This study identified several deficiencies in Chinese life education MOOCs. The general quality of the course is substandard, with several courses deficient in fundamental components, including course descriptions, essential instructional resources, and economic and technical support. Secondly, the convergence of course content is evident, with the significant overlap among certain courses, which fails to meet the individualized learning requirements of diverse students. Third, the courses lack opportunities for student feedback regarding the learning experience and do not facilitate comment programs to address students’ particular requirements. Studies indicate that student evaluations might enhance course quality and hence elevate student satisfaction (22). However, most of the courses do not activate the student comment channel, disregarding the individualized requirements of students.

The Global MOOC and Online Education Conference, themed “Reconstruction of Future Higher Education in the Age of Intelligence” held in the UK in 2024. It proposes three initiatives aimed at establishing MOOC standardization and normative development, enhancing collaborative efforts, and fostering a global online education community (23, 24). Consequently, the development of life education MOOCs should prioritize interactive feedback among learners and between learners and course developers. The functionalities of student inquiries and learning forums in MOOCs facilitate the sharing of perspectives and experiences among learners, fostering the interplay and synthesis of ideas, as well as the exchange and exploration of knowledge. Concurrently, the analysis of student performance and interactions within these forums allows for precise assessment of learning outcomes, providing significant insights for educational practices (25). Chinese MOOC platforms ought to draw insights from international counterparts, bolster collaboration with higher education institutions, integrate MOOCs into the curriculum, develop credit and degree certification frameworks, and further refine the certification system for MOOC learning (26).

### 4.3 Creating a distinctive MOOC format for life education in the era of online learning

This study found that life education MOOCs effectively encompass the themes of life health, life awareness, life value and life pursuit, facilitating profound contemplation of life and death among college students. MOOCs are extensively utilized in higher education owing to their flexibility in time and location, their ability to transcend regional limitations, and their facilitation of resource sharing, and they are progressively included into lifelong education (27). At present, life education MOOCs on the same platform in China adhere to identical assessment standards as other MOOCs, ensuring the efficacy of online learning. Nonetheless, life education encompasses extensive information and varied formats, and the grading evaluation approach based on scores is not conducive to accurately assessing students’ learning outcomes. The 2024 World Conference on Digital Education in China, themed “Artificial Intelligence and Digital Ethics,” emphasized that digital education and artificial intelligence education represent the future trajectory of educational development (23, 24). In recent years, data mining of MOOCs has garnered scholarly interest, with numerous researchers examining forum data on MOOC platforms (25), which facilitates student interaction for learning course content, posing inquiries, discussing specific topics, and sharing opinions and experiences.

Therefore, life education MOOC should conform to the online education background, fully apply big data and cloud computing to accurately analyze individual learning needs (28). They should gather and evaluate students’ classroom behaviors, learning conditions, and needs in real time, promote timely submission of feedback and learning summaries by students, and establish a personalized and intelligent online course framework for life education, thereby fostering students’ motivation to persist in their learning endeavors (29). Furthermore, we can pursue the trend of Virtual Reality (VR) education (30) by developing a VR system that allows real-time experiences of significant life processes, such as ‘dying and death, ‘thereby providing students with immersive life education, enhancing the learning experience, and elevating the quality of life education MOOCs.

## 5 Strengths and limitations

The strength of this study is that it investigates the current status of 129 life education MOOCs in China, focusing on course source and distribution, operational aspects, fundamental course information, and quality assessment, while also comparing them with overseas platforms.

The limitation of this study is that it is a retrospective study and the five MOOC platforms selected represent only some of the courses and the selection of five MOOC sites, which may not encompass all high-quality MOOCs for life education. Moreover, the accessibility of data from online platforms precluded the evaluation of student feedback, interactions, and assignment completion; hence, future researchers should augment the sample size to get more comprehensive results.

## 6 Conclusion

Life education is one of the important component of collegiate education. This study provides a comprehensive analysis of the characteristics of MOOC development in life education inside Chinese higher education institutions. Chinese life MOOCs offer abundant course resources, extensive subject coverage, and emphasize ideological education and the promotion of humanistic values. However, there are still problems such as insufficient personalized design, ambiguous course evaluation system, significant disparities between the East and the West. In the future, we must adhere to the evolving trends of artificial intelligence and online education, consider the physical and mental developmental characteristics of modern university students, develop high-quality courses, bridge the divide between Eastern and Western regions, and engage with international platforms to disseminate scientific and superior life education MOOCs globally.

## Data Availability

The original contributions presented in the study are included in the article/supplementary material, further inquiries can be directed to the corresponding author.

## Footnotes

^1^ sohu.com/a/754395783_120491808

^2^ zhihuishu.com

^3^ xueyinonline.com

^4^ icourse163.org

^5^ vocational.smartedu.cn

^6^ xuetangx.com

^7^ coursera.org

^8^ edx.org/school/usmx

^9^ qualitymatters.org
